# Nanotechnological Approaches to Enhance the Potential of α-Lipoic Acid for Application in the Clinic

**DOI:** 10.3390/antiox13060706

**Published:** 2024-06-09

**Authors:** Chiara Bellini, Fabrizio Mancin, Emanuele Papini, Regina Tavano

**Affiliations:** 1 Department of Biomedical Sciences, University of Padova, Via U. Bassi 58/b, 35121 Padova, Italy; chiara.bellini@ttk.elte.hu (C.B.); emanuele.papini@unipd.it (E.P.); 2 Department of Chemical Sciences, University of Padova, Via F. Marzolo 1, 35121 Padova, Italy; fabrizio.mancin@unipd.it

**Keywords:** α-lipoic acid, nanoparticles, antioxidant, cytotoxicity, poly(α-lipoic acid)

## Abstract

α-lipoic acid is a naturally occurring compound with potent antioxidant properties that helps protect cells and tissues from oxidative stress. Its incorporation into nanoplatforms can affect factors like bioavailability, stability, reactivity, and targeted delivery. Nanoformulations of α-lipoic acid can significantly enhance its solubility and absorption, making it more bioavailable. While α-lipoic acid can be prone to degradation in its free form, encapsulation within nanoparticles ensures its stability over time, and its release in a controlled and sustained manner to the targeted tissues and cells. In addition, α-lipoic acid can be combined with other compounds, such as other antioxidants, drugs, or nanomaterials, to create synergistic effects that enhance their overall therapeutic benefits or hinder their potential cytotoxicity. This review outlines the advantages and drawbacks associated with the use of α-lipoic acid, as well as various nanotechnological approaches employed to enhance its therapeutic effectiveness, whether alone or in combination with other bioactive agents. Furthermore, it describes the engineering of α-lipoic acid to produce poly(α-lipoic acid) nanoparticles, which hold promise as an effective drug delivery system.

## 1. Introduction

### 1.1. Chemical Structure of α-Lipoic Acid

α-lipoic acid, also known as thioctic acid, is a naturally occurring compound with potent antioxidant properties. 

**Figure 1:**
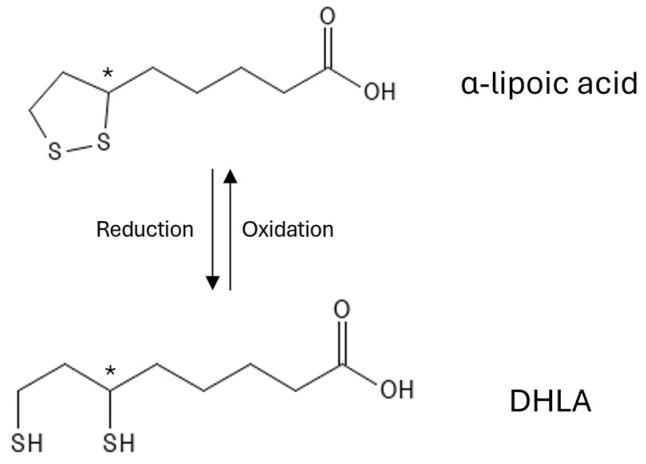
Structure of α-lipoic acid and its reduced form dihydrolipoic acid (DHLA). The asterisk (*) indicates the chiral center of α-lipoic acid and DHLA.

Structurally, it comprises three components: a five-atom dithiolate ring, an alkyl chain, and a terminal carboxylic acid (see Figure 1). The chiral centre at position C6 results in two enantiomers, R(+)-α-lipoic acid, the naturally available form, and S(-)-α-lipoic acid, which can be synthetically obtained [1].

R(+)-α-lipoic acid is generally recognized to exhibit superior activity compared to S(-)-α-lipoic acid [2,3,4,5,6], even as α-lipoic acid is usually marketed as a racemic mixture containing both enantiomers. To date, the extent to which each enantiomer contributes to α-lipoic acid pharmacological and toxicological effects is not fully understood. While it was proposed that S(-)-α-lipoic acid does not exhibit significant biological side effects [1,2,3,4,5,6,7], some studies indicated that it could potentially hinder the activity of the R(-)-enantiomer [8,9,10].

### 1.2. Antioxidant Activity of α-Lipoic Acid

The sulfur atoms within the α-lipoic acid ring play a crucial role in exerting antioxidant activity. The oxidised form (α-lipoic acid, ALA) and its reduced derivative (dihydrolipoic acid, DHLA) function as a potent redox couple, participating in different aspects of the antioxidant defence system (see Figure 1).

α-lipoic acid exerts its antioxidant activity through multiple mechanisms, including radical scavenging, the regeneration of other antioxidants, metal chelation, and signalling regulation. 

ALA and DHLA can neutralise a variety of reactive oxygen species (ROS) and reactive nitrogen species (RNS), thereby reducing oxidative stress and damage. Different mechanisms have been proposed for the free-radical scavenging activity of α-lipoic acid [11]. ALA was suggested to work through a proton-loss electron transfer mechanism, while DHLA exhibits its activity by single electron transfer followed by proton transfer, or hydrogen atom transfer. In the latter, the ideal spacing between the sulfhydryl and carboxylate groups, determined by the optimal length of the alkyl chain, facilitates the efficient trapping of radicals and the transfer of hydrogen atoms [12]. 

Generally, antioxidants are categorised based on their solubility characteristics. Water-soluble antioxidants like vitamin C are effective in aqueous environments, while hydrophobic antioxidants like vitamin E prefer lipid-rich environments. Due to its amphiphilic nature, α-lipoic acid represents a unique universal antioxidant, exhibiting activity in aqueous and lipid-based environments, thus protecting a wide range of cellular structures and biomolecules. 

The capacity of α-lipoic acid to form complexes with redox-active metals presents a dual benefit. On the one hand, it acts as a chelator for heavy metals like mercury, iron, cadmium, lead, and copper, mitigating their toxicity [13,14,15]. On the other hand, its ability to bind metals, especially gold, has been used to create innovative nanostructures for targeted drug delivery, imaging, and theranostic applications [16].

One distinctive feature of α-lipoic acid is its ability to regenerate the reduced form of either non-enzymatic antioxidants, such as vitamins C and E, glutathione and cysteine, or enzymatic antioxidants, like the coenzyme Q10 [17]. By recycling other antioxidants, α-lipoic acid actively contributes to maintaining the antioxidant network and the cellular redox equilibrium. 

Moreover, α-lipoic acid acts as a modulator of proteins involved in cell signalling and transcription through the thiol/disulfide exchange mechanism [18,19,20]. As a result, it has been proposed to influence different aspects, including cell growth and death regulation, inflammatory responses, and glucose and lipid metabolism.

### 1.3. α-Lipoic Acid as a Therapeutic Agent

The extensive range of protective mechanisms exhibited by α-lipoic acid has attracted interest regarding its potential use as a therapeutic agent. Specifically, it has gained particular attention in clinical conditions involving oxidative stress and inflammation, preserving various cell types and organs from oxidative damage. 

Due to its role in contributing to cellular energy homeostasis, α-lipoic acid has proven beneficial against insulin resistance, metabolic syndrome, type 2 diabetes, and diabetes-related complications, such as diabetic neuropathy and nephropathy [21,22,23,24,25,26]. Notably, α-lipoic acid demonstrates nephroprotective effects and holds promise in liver disease prevention due to its rapid hepatic metabolism [27,28,29,30,31,32,33]. Several studies have described the cardiovascular benefits of α-lipoic acid administration [34,35,36,37,38]. Moreover, its ability to cross the blood–brain barrier has shown potential in protecting against neurodegenerative diseases, such as multiple sclerosis and Alzheimer’s and Parkinson’s diseases [39,40,41]. Also, α-lipoic acid has been suggested to have immunomodulatory activity on both innate and adaptive immune cells, down-regulating the proinflammatory markers and reducing the inflammatory response [11,42]. The topical application of α-lipoic acid has proved effective in skin-related conditions, such as ageing and wound healing, in which its antimicrobial properties may ensure a safe repair of skin damage [11,43,44,45,46,47,48]. The use of α-lipoic acid was also investigated in cancer research and it was described as having antiproliferative and antimetastatic activity in different cancer models [49,50,51,52,53].

The unique structure of α-lipoic acid not only makes it a versatile antioxidant but also a promising scaffold for derivatization, providing opportunities for the creation of novel therapeutic agents. From a chemical perspective, the dually modifiable structure of α-lipoic acid allows the functionalization of the dithiolane ring and the carboxylic acid. This feature enables the properties and activity of either the ligands or α-lipoic acid itself to be customised.

### 1.4. Limitations and Drawbacks in the Therapeutic Use of α-Lipoic Acid

While showing diverse beneficial effects, α-lipoic acid and its therapeutic usage exhibit some drawbacks. 

α-lipoic acid is mainly administered orally in solid or liquid form. However, the oral administration of α-lipoic acid results in a limited pharmacokinetic profile, characterised by a short half-life, reaching the maximum plasma concentration in approximately 30 min, and reduced bioavailability (about 30%) [54,55,56,57,58]. This drawback becomes especially significant when administering α-lipoic acid as a racemic mixture. In fact, stereochemistry has been proved to notably affect the absorption, distribution, degradation, and elimination of lipoic acid enantiomers. R-(+)-α-lipoic acid has higher bioavailability than the S-(+) enantiomer, potentially because of the different mechanisms of absorption in the gastrointestinal tract [5,8,54,55,56,57,59,60]. The suboptimal pharmacokinetics of α-lipoic acid have been attributed to its reduced solubility in water and acidic environments and consequent limited gastric stability, and its high first-pass hepatic metabolism [8]. As a result, it needs appropriate formulation to optimise its therapeutic efficacy when used as a supplement or medication [59,61,62,63].

While the prolonged use of α-lipoic acid has been demonstrated to be well-tolerated among healthy individuals [64,65] and it is generally considered not toxic [66,67], registered adverse effects have been correlated with the use of high α-lipoic acid doses [68,69,70], such as gastrointestinal side effects such as stomach discomfort, nausea, or diarrhoea [71]. Moreover, α-lipoic acid at a high dosage negatively affected the liver enzyme activity and induced liver damage in mice and monkeys [66,72]. Also, its supplementation may lower blood sugar levels in genetically predisposed individuals, which can be problematic for diabetic individuals [73]. Finally, α-lipoic acid may interact with certain medications, including chemotherapy drugs, thyroid medications, and drugs that lower blood sugar levels. 

Some in vitro and in vivo studies have indicated the pro-oxidant potential of α-lipoic acid, whether in the oxidised form or DHLA [73,74,75]. The result of this effect with either beneficial or detrimental outcomes depends on the specific physiological circumstances, although the pro-oxidant activity of α-lipoic acid has been associated with cardiovascular, renal, and liver damages [73,74,75].

From the chemical point of view, α-lipoic acid exhibits high sensitivity to various environmental factors. It gradually polymerizes, increasing the temperature, and forming an elastic compound [76,77]. Moreover, it is photosensitive, and exposure to UV light can impair the hindered dithiolane ring, leading to undesired polymerization [78]. α-lipoic acid is poorly soluble in water, but soluble in organic solvents. However, the drying process has been suggested to impact its stability. Specifically, Brown and Edwards [79] reported that solvents with easily extractable hydrogens or chlorine atoms minimise α-lipoic acid polymerization in solution. Thus, solvent evaporation at high temperatures and under reduced pressure could reduce the stabiliser effect of hydrogen/chlorine atoms. 

**Figure 2:**
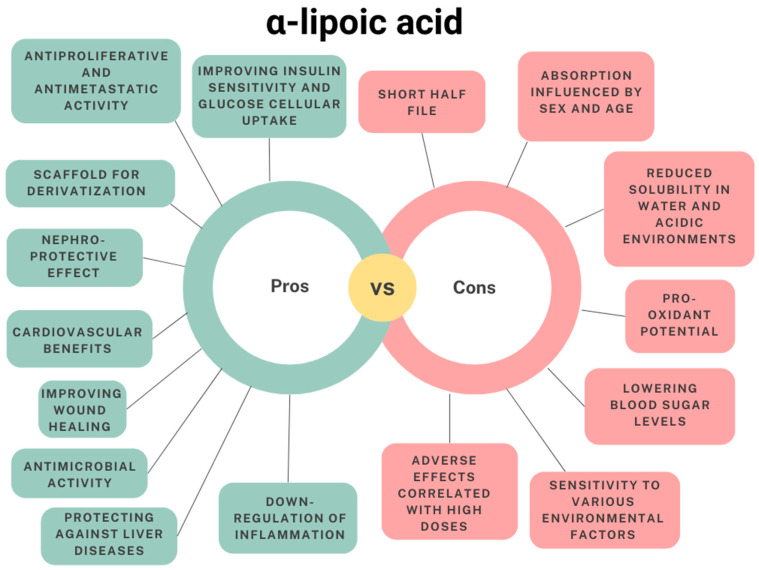
Beneficial aspects and limitations of α-lipoic acid.

Limitations and benefits of the use of α-lipoic acid are summarised in Figure 2.

### 1.5. Nanomedicine as a Strategy to Improve α-Lipoic Acid

Nanomedicine, the “application of nanotechnology for treatment, diagnosis, monitoring, and control of biological systems” [80,81,82], has raised great expectations [83]. Therapeutic and diagnostic nanoagents (nanotheranostics) may improve the pharmaceutical activity while reducing side effects [84], thanks to multiple functionalisations. Moreover, coating with polymers, like polyethylene glycol, reduces the binding of plasma opsonins, making nanoparticles invisible to macrophages’ clearance [85,86,87]. 

Nanoparticles with antioxidant properties have recently emerged as possible therapeutic agents for controlling neurodegenerative diseases [88] and cardiovascular [89] and pulmonary diseases [90], and in wound healing [91] and the treatment of cancer [92]. 

This review aims to outline the application of α-lipoic acid to the nanomedicine field and to explore the advantages associated with its inclusion in nanoplatforms. Indeed, this work describes the strategies employed to increase the shelf life of α-lipoic acid through encapsulation in different nanocarriers, as well as studies utilising mixtures of α-lipoic acid and nanoparticles to enhance the biocompatibility or therapeutic activity of the nanoparticles. Moreover, new frontiers of nanocomposites obtained with poly(α-lipoic acid) will be discussed. 

## 2. Enhancement of α-Lipoic Acid Properties and Activity When Encapsulated in Nanoparticles

**Figure 3:**
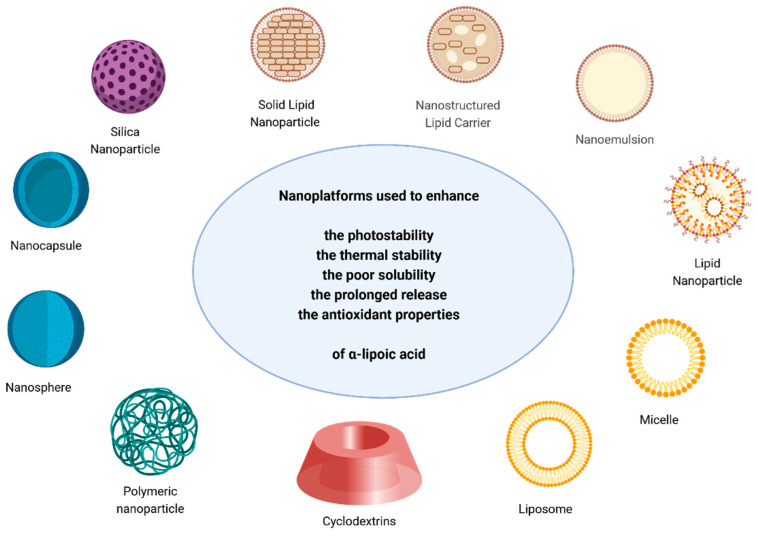
The different nanoplatforms used to encapsulate α-lipoic acid. Created with BioRender.com (accessed on 3 June 2024).

As described in the introduction, the primary challenges associated with the use of α-lipoic acid are its short half-life, limited bioavailability, and poor solubility. This section describes several strategies employed to address these drawbacks and improve α-lipoic acid properties by encapsulating the free molecule into different nanotechnological platforms [93]. The delivery systems established are visualised in Figure 3, while Table 1, at the end of this section, summarises the nanotechnological strategies described throughout the text.

### 2.1. Silica Nanoparticles

Silica nanoparticles represent suitable candidates to overcome several challenges faced by bioactive compounds, including hydrolysis, solubility concerns, and susceptibility to light and heat degradation [92]. 

To enhance the α-lipoic acid photo- and thermal stability, Dolinina et al. synthesised novel mesoporous silica-based nanocomposites using the widely applied sol–gel method [93]. Two different final products were proposed for α-lipoic acid thermal- and photodegradation, namely polymeric α-lipoic acid and DHLA, respectively. α-lipoic acid was stabilised by the interaction with the silica matrices through H-bonds and electrostatic and hydrophobic forces. As a result, its thermal degradation decreased when compared to the free molecule. As regards UV-induced degradation, the photodegradation rates were comparable to the free α-lipoic acid. The authors suggested that the significant amount of water contained in the nanocomposites, being a suitable hydrogen atom source, favoured the formation of DHLA. Also, the stability of the nanocomposites and the release of α-lipoic acid was investigated, mimicking the pH of digestive fluids. The silica-based composites displayed a release behaviour close to the zero-order kinetic, sustained a plasma concentration of α-lipoic acid comparable to clinically available oral formulations, and preserved its antioxidant activity at all pH values for up to 24 h [94].

### 2.2. Lipid-Based Nanoparticles (Lipid Carriers, Solid Lipid Nanoparticles, Nanoemulsions, Liposomes, Micelles)

Encapsulation into lipid nanoparticles can overcome the poor solubility of compounds in aqueous media, and their susceptibility to degradation when exposed to oxygen in water. Moreover, lipid nanoparticles have been shown to sustain the release of different drugs, increasing the therapeutic effect and bioavailability and enhancing their biocompatibility. 

Platforms belonging to this category include nanostructured lipid carriers, solid lipid nanoparticles, nanoemulsions, liposomes, lipid nanocapsules, and micelles. They are composed of lipids comprising fatty acids, fatty alcohols, glycerides, and waxes, along with stabilising agents like surfactants or polymeric coatings [95]. 

While solid lipid nanoparticles are formed from solid lipids stabilised by surfactants, nanostructured lipid carriers include both solid and liquid lipids in their matrix. They represent the next generation lipid nanoparticles, designed to overcome the limitations of conventional colloidal carriers, like emulsions, liposomes, and polymeric nanoparticles [96,97]. 

Indeed, when α-lipoic acid was formulated into nanostructured lipid carriers, solid lipid nanoparticles, and nanoemulsions, the former two displayed a prolonged release of α-lipoic acid compared to nanoemulsions. They also proved high entrapment efficiency and stability over time, and exhibited antioxidant activity comparable to pure α-lipoic acid [98]. 

Conjugation with stearylamine was used to stabilise α-lipoic acid and synthesise solid lipid nanoparticles, and was then investigated as a delivery system for the chemotherapeutic tamoxifen. The formulation was proved to stabilise α-lipoic acid, which exerted its antioxidant activity, restoring the antioxidant protein network. Moreover, it efficiently enhanced the bioavailability of tamoxifen [99]. Also, solid lipid nanoparticles, co-delivering docetaxel and α-lipoic acid, were demonstrated to have enhanced efficacy against murine mammary carcinoma cells and human breast adenocarcinoma cells. The nanoformulation containing the chemotherapeutic agent and the antioxidant molecule showed increasing cytotoxic effects and significantly higher uptake efficiency compared to free drugs or single drug-loaded solid lipid nanoparticles [100].

Wang et al. synthesised nanostructured lipid carriers using the biodegradable components phosphatidylcholine, stearic acid, oleic acid, and glyceryl monostearate, with the aim of incorporating α-lipoic acid and improving its stability and solubility. The authors found that the obtained lipid carriers were not cytotoxic in vitro, retained α-lipoic acid, and released it within 72 h [101]. 

Phosphatidylcholine was also employed in the formulation of both nanoemulsions and liposomes containing α-lipoic acid. While both nanosystems efficiently entrapped α-lipoic acid, nanoemulsions exhibited a slower release of α-lipoic acid compared to phosphatidylcholine liposomes. The authors proposed that the slower release from nanoemulsions could be attributed to their multiple-layer structure, whereas liposomes, being primarily single-layered particles, facilitated a faster release. Thus, nanoemulsions might represent a more suitable formulation for α-lipoic acid, prolonging the drug’s action [102]. 

Different oil-in-water nanoemulsions were also investigated for the co-delivery of α-lipoic acid and vitamin B. Specifically, the influence of using different oils (castor oil and sunflower oil) and agitation methods on the properties of the obtained emulsions was evaluated, including metrics of colloidal stability, encapsulation efficiency and drug release. The formulation prepared by the widely applied solvent displacement method and using castor oil under magnetic stirring resulted in the best colloidal properties, highest thermal and pH stability, and higher encapsulation and release rates [103]. In another study, pumpkin oil was used to solubilize α-lipoic acid and develop a self-nanoemulsifying drug delivery system, stabilised by Tween 80 polyethylene glycol 200. The formulation demonstrates higher protective effects against gastric ulcer models compared to the free α-lipoic acid [104].

Phophatidylcholine-based liposomes were prepared to improve the stability and absorption of α-lipoic acid. The formulation proved to be stable, enhanced the oral absorption and bioavailability of α-lipoic acid, and improved hepatoprotection in hepatic injury model rats [105]. Other authors have synthesised new liposomes, consisting of phosphatidylcholine and curcumin, in which α-lipoic acid has been encapsulated; using these liposomes, they demonstrated a high encapsulation efficiency of lipoic acid and its slow release, which countered cisplatin-induced toxicity in HEI-OC1 cells, a cell line utilised as a model for auditory cells [106].

Polymers can be used to stabilise different platforms, including emulsions, liposomes, and micelles. Huang et al. developed an advance system for preparing a multilayer emulsion. A primary oil-in-water emulsion, stabilised by Tween 20 and phosphatidylcholine, was used to encapsulate α-lipoic acid and linseed oil. Subsequently, a chitosan coating was applied to improve the thermostability of the oil droplets and to address the limited capacity of emulsions to counteract oxidative stress, which could potentially affect the stability of both encapsulated molecules. The system demonstrated a higher thermal stability than the primary emulsion and limited lipolysis of linseed oil in vivo, and inhibited the degradation of α-lipoic acid [107]. The use of a chitosan coating also proved efficient in stabilising liposomes co-encapsulating α-lipoic acid and Coenzyme Q10 with the purpose of improving the solubility and stability of both molecules for transdermal application. It was found that chitosan interacted with α-lipoic acid and Coenzyme Q10 via H-bonds and ionic forces, promoting the permeation and accumulation of both molecules in rabbit skin. Moreover, the system demonstrated higher scavenging activity compared to free α-lipoic acid and Coenzyme Q10 [108]. α-lipoic acid was also incorporated into micelles composed of a copolymer (methoxypoly(ethylene glycol) and poly(caprolactone)). This incorporation led to an enhanced loading capacity of the micelles for IR780, a near-infrared dye for imaging and photothermal therapy, while also preserving their loading capacity for docetaxel, a chemotherapeutic agent [109]. Pluronic nanoparticles were prepared by the oil-in-water emulsion method, encapsulating α-lipoic acid into a soybean oil core and using Pluronic F127 to form the hydrophilic shell. When administered in the mouse middle ear cavity, the system was demonstrated to preserve hearing ability after ototoxicity induction, and could be delivered in a more effective way with respect to free α-lipoic acid [110].

### 2.3. Nanocapsules and Nanospheres

Nanocapsules are composed of a polymeric shell surrounding an oily core, in which the drug may either dissolve within the core or adhere to the polymeric wall. In contrast, nanospheres lack an oily component and instead constitute a polymeric matrix, in which the drug can be uniformly distributed or adsorbed.

α-lipoic acid was encapsulated in lipid-core nanocapsules with triglycerides of capric and caprylic acid, sorbitan monostearate, and poly (ε-caprolactone) as main components, and stabilised by polysorbate 80. The encapsulation counteracted α-lipoic acid’s undesired pro-oxidant activity, and contributed to the wound healing process, although to a lesser extent than lipoic acid alone [43]. Also, Xia et al. encapsulated α-lipoic acid into lipid nanocapsules containing triglycerides of capric and caprylic acids and phosphatidylcholine as a surfactant. The capsules proved to be stable and have high encapsulation efficiency and drug loading. Moreover, antioxidant activity remained intact within the nanocapsules, and sustained release of α-lipoic acid was achieved [111]. Interestingly, a dual stabilising effect of α-lipoic acid and the antioxidant resveratrol was observed when co-encapsulated into lipid-core nanocapsules. The co-encapsulation was shown to enhance the stability, photostability, and antioxidant properties of both compounds. When α-lipoic acid was encapsulated alone, it exhibited rapid release, consistent with previous findings [112]; however, when co-encapsulated with resveratrol, the release rates of both compounds decreased [113]. 

Nishiura et al. exploited the self-assembly properties of α-lipoic acid, along with the non-ionic surfactant polyoxyethylene (20) stearylether, to create a nanosphere matrix encapsulating α-lipoic acid. The encapsulation improved the stability and bioavailability of α-lipoic acid by placing its hydrophobic moiety inside the nanocapsule, thereby protecting the unstable disulfide bond from degradation [77]. The same method was used to encapsulate α-lipoic acid and test its antitumor effects in a mouse model of Ehrlich solid tumour. Both free and encapsulated α-lipoic acid significantly reduced oxidative stress, but encapsulated α-lipoic acid remained stable and in circulation for longer periods of time [114]. Also, EL-Gebaly et al.’s approach was used to investigate the beneficial effects of encapsulated α-lipoic acid against 99mTc-MIBI-induced injury, showing significant protective effects and suggesting its efficacy as a radioprotector [115]. Kubota and collaborators used a similar approach by mixing α-lipoic acid with the polyoxyethylene (20) stearylether to improve its skin permeation. The results confirmed that nanoencapsulation is a suitable method for the efficient topical administration of α-lipoic acid [116]. 

### 2.4. Polymeric Nanoparticles

Natural polymers have frequently been used to develop biodegradable and biocompatible delivery systems.

Gogoi et al. co-loaded α-lipoic acid and curcumin into chitosan nanoparticles to improve the limited solubility and bioavailability of both drugs. They investigated the in vitro activity of the nanosystem in a breast cancer cell model, highlighting the synergistic activity of curcumin and α-lipoic acid in inhibiting tumour cell growth and inducing tumour cell death [117]. Also, the bioavailability and stability of α-lipoic acid formulated into chitosan nanoparticles and solid lipid nanoparticles was compared [118]. Both systems encapsulated α-lipoic acid effectively, demonstrated high stability, and counteracted the oxidative stress and neurotoxicity induced by aluminium chloride in rats. However, as expected, the effect was superior when α-lipoic acid was loaded into solid lipid nanoparticles compared to chitosan nanoparticles. 

To improve its skin delivery, α-lipoic acid was loaded into liquid crystalline nanoparticles. The surface of the platform was modified by lipopolymers composed of Polyethylene glycol (PEG) and 1,2-dioleoyl-sn-glycero-3-phosphoethanolamine, and functionalized with the epidermal growth-factor-receptor-targeting D4 peptide, and the HIV-1-derived TAT cell-penetrating peptide. The system demonstrated a high drug encapsulation rate and released the α-lipoic acid into the deepest layers of the epidermis, where it counteracted the oxidative stress and inflammation after exposure to UVB light [119].

The use of α-lipoic acid is also interesting in suppressing appetite. Traditional formulations (salt or micronized crystals) had poor absorption and a short plasma half-life, not sufficient to suppress appetite. Park and collaborators introduce a novel α-lipoic acid nanoparticulate formulation developed through nano-comminution, employing different polymeric stabilisers (hydroxypropyl cellulose, Pluronic^®^ F127, and polyvinylpyrrolidone). Interestingly, they found that nanoparticle powder formulations, when compared to nanosuspension formulations, were endowed with an improved efficacy in reducing food intake [120].

### 2.5. Cyclodextrins

Cyclodextrins (CDs) are cyclic oligosaccharides derived from the enzymatic degradation of starch which typically comprise six (α-), seven (β-), or eight (γ-) glucose units linked through α-1,4 glycosidic bonds. They have a toroidal-like shape characterised by a hydrophilic outer surface and a more hydrophobic central cavity. This distinctive arrangement enables them to interact with molecules of appropriate size and geometry, and form complexes with the entire molecule or part of it. They are frequently used as excipients in pharmaceutical formulations to increase the aqueous solubility of poorly soluble active substances, improving the stability and bioavailability of drugs [121,122,123].

α-lipoic acid has been proven to be complexed by α-, β-, and γ-CDs [124,125], and in particular the R-(+)-α-lipoic acid complex with γ-CD exhibited biocompatibility and stability towards heat, humidity, and low pH [76]. γ-CD significantly improved the bioavailability of R-(+)-α-lipoic acid in rats by enhancing its intestinal absorption [126]. As a result, the R-(+)-α-lipoic acid/γ-CD complex exhibited a 2.2 times higher plasma concentration compared to free α-lipoic acid, after oral administration. This evidence was confirmed by a recent clinical trial in healthy volunteers, where the bioavailability of the R-(+)-α-lipoic acid/γ-CD complex demonstrated results 2.5 higher than the non-complexed R-(+)-α-lipoic acid [127]. 

**Table 1:** Nanotechnological strategies used to improve α-lipoic acid characteristics and efficacy.

Nanoplatform	Goal	Reference
Silica nanoparticles	Stabilise	[93,94]
Lipid-based nanoparticles	Prolong the release	[98,100,101,102,103,106,108,110]
	Stabilise	[99,103,107,110]
	Improve solubility	[104]
	Improve bioavailability	[105]
	Improve antioxidant activity	[108]
Nanocapsules and nanospheres	Counteract pro-oxidant activity	[43]
	Preserve antioxidant activity	[111]
	Improve the stability	[114]
	Prolong the release	[111,113,114]
Polymeric nanoparticle	Improve the solubility and bioavailability	[118,119,120]
	Improve the release	[120]
Cyclodextrins	Improve the stability and bioavailability	[76,126,127]

## 3. Exploring the Role of α-Lipoic Acid in Therapeutic Nanoplatforms

α-lipoic acid has been studied in numerous works in combination with several types of nanoparticles. The formulations have been shown to enhance either the effects of nanoparticles or α-lipoic acid antioxidant properties, both in cellular systems and in mouse models of different pathologies. 

### 3.1. Renewing Tissues: Nanoparticles and α-Lipoic Acid in Regenerative Medicine

The application of nanotechnology to non-healing wounds aims to facilitate healing and tissue repair and avoid complications like infections. 

The potential benefits of therapies based on a mixture of nanoparticles and antioxidant agents on tissue regeneration have been demonstrated by Leu et al. The topical application of a mixture containing gold nanoparticles, the antioxidant epigallocatechin gallate, and α-lipoic acid significantly enhanced the proliferation and migration of skin cells and accelerated wound healing in mice [128]. The same combination was also investigated in the treatment of diabetic ulcers, reducing the expression of the receptor for advanced glycation end-products, enhancing angiogenesis, and exerting anti-inflammatory effects [129]. 

Moreover, wound healing was improved by the treatment with α-lipoic acid conjugated with hexagonal boron nitride and boron carbide nanoparticles, exhibiting regenerative and antioxidant properties. α-lipoic acid has also been demonstrated to improve the antimicrobial properties of boron nitride and boron carbide nanoparticles against *Staphylococcus aureus* and *Escherichia coli* strains [130]. 

Finally, α-lipoic acid-capped silver nanoparticles introduced into alginate-based aerogels demonstrated improved biological properties, including higher antioxidant capacity and anti-inflammatory activity, compared to a commercial dressing made of alginate containing colloidal silver [131].

### 3.2. Exploring the Use of α-Lipoic Acid-Containing Nanoparticles in Tumour Fighting

In cancer nanomedicine, α-lipoic acid has found application in three primary aspects: (1) the exploitation of its intrinsic antitumor activity when nanoformulated; (2) the development of tumour environment-responsive nanoparticles, taking advantage of the characteristics of its disulfide bond; (3) its action as a linker to enhance the binding strength of tumour-targeting ligands on the surface of nanoparticles.

As reviewed elsewhere, α-lipoic acid has different powerful effects against cancer cells, such as inducing apoptosis and suppressing proliferation, and cancer stemness. Moreover, it shows potential in mitigating chemotherapy-induced side-effects and overcoming chemoresistance [49,132]. 

α-lipoic acid can be nanoformulated and investigated for its potential effects against tumour cells. For example, Usama et al. proposed the synergistic activity of α-lipoic acid with simvastatin, a statin with direct cytotoxic activity against different types of tumour cells. The synthesised α-lipoic acid-simvastatin nanoparticles demonstrated a significant increase in the cellular uptake and cytotoxicity of the statin in breast carcinoma cells [133].

The disulfide bond of α-lipoic acid can be exploited to prepare stimuli-responsive nanoparticles, specifically systems that respond to the reducing environment found in tumour cells. In fact, it is well known that the concentration of glutathione in its reduced form (GSH) is significantly higher in cancer cells, and it can trigger the reduction of the α-lipoic acid dithiolane ring, facilitating the release of the chemotherapeutics. 

For example, α-lipoic acid was esterified to phosphatidylcholine to produce stimuli-responsive cross-linked liposomes for the intracellular delivery of doxorubicin. The system demonstrated a high serum stability and reduced-responsive drug release, resulting in an in vitro anticancer activity comparable to the free doxorubicin. Moreover, the liposomal formulation improved the pharmacokinetic profile of doxorubicin, which was maintained in the blood circulation for a longer time [134]. Also, α-lipoic acid was conjugated to a tocopheryl moiety to produce stimuli-responsive nanovesicles designed for the delivery of doxorubicin. The nanovesicles efficiently encapsulated doxorubicin, and released it in a glutathione-responsive manner. Moreover, the system proved to be biocompatible and to have an anticancer activity superior to doxorubicin alone in drug-resistant cancer cells [135]. Also, α-lipoic acid was conjugated to xylan to produce redox-sensitive nanoparticles aimed to deliver the anticancer drug niclosamide. The platform exhibited good stability and biocompatibility as well as higher anticancer activity compared to the free drug [136].

Additionally, dual targeting delivery systems have been developed, combining the stimuli-responsive behaviour of α-lipoic acid and other tumour-specific targets or tumour-specific environmental conditions (see Table 2). In this category, several works exploit the concept of charge-reversible nanoplatforms, capable of adjusting their surface charge according to the surrounding environment [137]. They exhibit either a negative or a neutral charge under physiological pH conditions to prevent adsorption to serum proteins that can induce opsonization, complement activation, and rapid clearance. To achieve this feature, specific functional groups, such as the imidazole moiety and maleic anhydride [138,139,140,141], or coatings like polyethylene glycol, have been introduced [138,141,142]. Consequently, charge-reversible nanosystems exhibit enhanced plasma stability and can accumulate in tumour tissues. Once it has reached the tumour microenvironment, the acidic pH triggers the charge switch and the positively charged surface facilitates the cellular internalisation of the delivery platform.

In other works, tumour-specific ligands were introduced to improve the targeting of the delivery systems, such as binders of the tumour-overexpressed receptors [138,143,144,145,146,147,148]. 

After internalisation, drug release was achieved by the activity of high glutathione intracellular concentration on α-lipoic acid.

**Table 2:** α-lipoic acid-based dual responsive drug delivery systems in cancer treatment.

Tumour Target	Molecule	Nanoplatform	Goal	Reference
Acidic pH and Overexpressed ASGPR *	Dimethylmaleic acid—PEG and lactobionic acid	Polymeric nanoparticle	Delivery of Doxorubicin	[138]
Overexpressed ASGPR and folate receptor	Pullulan and folic acid	Polymeric nanoparticle	Delivery of Paclitaxel	[148]
Overexpressed folate receptor	Folic acid	Silica hybrid Magnetic nanoparticles	Delivery of Doxorubicin	[143]
Acidic pH	Histidine	Polymeric nanoparticle	Delivery of Doxorubicin	[139]
	Dimethylmaleic anhydride	Polymeric nanoparticle	Delivery of Doxorubicin	[140]
	Histidine—PEG	Liposomes	Delivery of VEGF *** siRNA and Etoposide	[141]
	PEG	Albumin-based nanocarrier	Delivery of Doxorubicin	[142]
CD44 receptor overexpression	Hyaluronic acid	Polymeric nanoparticle	Delivery 17α-Methyltestosterone	[134]
	Hyaluronic acid	Polymeric nanoparticle	Delivery of Doxorubicin	[135]
Tumour esterase overexpression	Chlorambucil	Polymeric nanoparticle	Delivery of Doxorubicin and Chlorambucil	[136]
αvβ3 receptor overexpression	cRGD peptide **	Micelle	Delivery of Doxorubicin	[137]

* asialoglycoprotein receptor; ** cyclic arginyl-glycyl-aspartic acid peptide; *** vascular endothelial growth factor.

Thiols are commonly used as ligands for attaching drugs to the surface of nanoparticles, such as gold nanoparticles. Using linkers with multiple thiols, such as α-lipoic acid, increases the binding strength between the nanoparticles’ surface and the ligands. Also, α-lipoic acid’s carboxylic group can be directly functionalised with chemotherapeutics, spacers, and tumour-specific targets. The following works, mainly focused on the functionalisation of gold nanoparticles, describe the use of α-lipoic acid for these purposes. Ghorbani et al. used α-lipoic acid as a linker to attach curcumin to the surface of Fe_3_O_4_, resulting in a novel, particularly potent medicinal compound with increased cytotoxicity compared to curcumin alone [149]. Also, promising antimetastatic activity was shown by linking a tumour-associated carbohydrate antigen to gold nanoparticles through an analogue of α-lipoic acid, the iso-α-lipoic acid. Moreover, the advantage of having an achiral linker as iso-α-lipoic acid lies in the simplified design, synthesis, and characterisation of the nanosystem [150]. α-lipoic acid was used to link folic acid and doxorubicin to the surface of gold nanoparticles to obtain a targeted delivery system for the chemotherapeutic drug. The approach resulted in enhanced cell penetration and the prolonged release of doxorubicin [151]. α-lipoic acid was used in the development of gold tumour-targeting nanoparticles capable of responding to the acidic intracellular environment and aggregating to amplify nanoparticle retention within tumour cells. Specifically, in the work of Cheng, α-lipoic acid was used to link a biotin-end-derived PEG moiety to the gold surface. As a result, the nanoplatform exhibited stealth properties and active targeting ability [152]. A similar approach was used to conjugate doxorubicin and anti-PD-L1 antibody on the gold nanoparticle surface, through an α-lipoic acid-PEG-*N*-hydroxysuccinimide linker. The thiol groups of α-lipoic acid interacted with the gold surface, while it formed an amide bond with the *N*-hydroxysuccinimide moiety binding the cancer-targeting ligands [153].

Wang and collaborators modified star poly(lactic-co-glycolic acid) ends with α-lipoic acid to obtain a new nanoparticle with enhanced loading capacity, reduced drug leakage, and efficient uptake in melanoma cells [154,155].

### 3.3. Exploring Miscellaneous Applications of Nanoparticles and α-Lipoic Acid

The combination of α-lipoic with nanomaterials, including metal and polymeric nanoparticles, was also investigated in other therapeutic areas. 

Indeed, Tudose et al. covalently immobilised α-lipoic acid onto the surface of silver nanoparticles–decorated silica nanoparticles, and they evaluated the antioxidant, cytotoxic, and antimicrobial activities of these new nanoparticles. They found that the functionalisation of silver nanoparticles–decorated silica nanoparticles with α-lipoic acid enhanced the specificity of the interaction with mammalian cell lines and antioxidant activity, while reducing cytotoxicity, suggesting its potential for modulating the cell cycle to achieve desired therapeutic effects [156]. 

The mixture of α-lipoic acid with gold nanoparticles showed a superior neuroprotective effect compared to each compound alone in vivo, with the inhibition of brain damage induced by radiation in rats [157]. The combination of α-lipoic acid with PEGylated gold nanoparticles proved to counteract oxidative stress in osteoporosis development. α-lipoic acid-gold nanoparticles were biocompatible, and effectively removed reactive oxygen species and promoted osteoblast proliferation [158]. 

The impact of α-lipoic acid in combination with caffeine-loaded chitosan nanoparticles was evaluated against obesity and its hepatic and renal complications in rats. The treatment was shown to mitigate weight gain and improve hepatic and renal function, reducing biochemical markers and histopathological alterations [159]. The same nanosystem was also tested for its ability to mitigate cardiovascular complications induced by obesity in rats. Treatment with α-lipoic acid and caffeine-loaded chitosan nanoparticles alleviated cardiac complications and the cardioprotective effect of the combined treatment, was more evident than that of the two agents used individually [160]. A nanosphere matrix made of zein (a water-insoluble plant protein derived from maize) and α-lipoic acid was employed to encapsulate Vardenafil, a medication used for the treatment of erectile dysfunction [161]. α-lipoic acid has indeed previously been shown to significantly improve sexual function and quality of life in both animals and human investigations [162]. In the work of Ahmed [161], α-lipoic acid was stabilised by the interaction with the zein polymer; moreover, the bioavailability, delivery, and effects of Vardenafil were improved thanks to the nanoformulation.

## 4. Protective Role of α-Lipoic Acid against Nanoparticle Cytotoxicity

**Figure 4:**
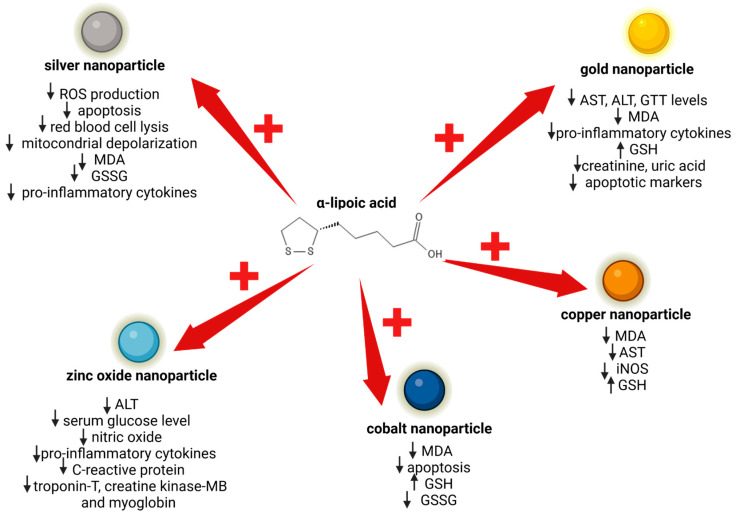
The impact of α-lipoic acid on mitigating the cytotoxic effects induced by various metal nanoparticles. ALT: alanine aminotransferase; AST: aspartate transaminase; MDA: malondialdehyde; GSSG: glutathione disulfide; GSH: glutathione; iNOS: inducible nitric oxide synthase. Created with BioRender.com (accessed on 3 June 2024).

Different studies have shown that α-lipoic acid can inhibit the intrinsic cytotoxicity of various types of nanoparticles. Two strategies have been used by researchers: on one hand, directly conjugating α-lipoic acid to the surface of the nanoparticle, and on the other hand, mixing α-lipoic acid with the nanoparticles before application. Using α-lipoic acid in conjunction with various types of nanoparticles has been demonstrated to limit many of the cytotoxic effects induced by the nanoparticles themselves (see Figure 4).

### 4.1. α-Lipoic Acid and Silver Nanoparticle Cytotoxicity

As is already known, silver nanoparticles possess potent antimicrobial activity, but they can exert cytotoxic effects [163,164]. Hajtuch et al., comparing uncoated silver nanoparticles with α-lipoic acid silver nanoparticles, found that silver nanoparticles coated with α-lipoic acid maintained their antimicrobial properties, while improving their biosafety. In fact, α-lipoic acid silver nanoparticles were found to be biocompatible with Huvec cells, red blood cells, and platelets [165]. Also, Cotton and collaborators mitigated silver nanoparticles’ toxic effects by capping them with α-lipoic acid. The protective effect of α-lipoic acid was investigated on human gingival fibroblasts in comparison to ionic silver and clinical antiseptics (chlorhexidine and silver diamine fluoride). The authors found that α-lipoic acid-capped silver nanoparticles exhibited notably lower toxicity than the clinically utilised controls, while keeping their antimicrobial properties intact [166]. In another study, the damage induced on rat testes by silver nanoparticles was attenuated by mixing them with α-lipoic acid [167]. The authors demonstrated that the co-treatment mitigated the biochemical, oxidative, and apoptotic changes induced by silver nanoparticles. These kinds of nanoparticles, when administered, can damage the blood–brain barrier function and induce neurotoxicity; the co-treatment with α-lipoic acid was demonstrated to be favourable in ameliorating the neurotoxic side effects of silver nanoparticles by reducing the induced apoptosis, inflammation, and oxidative stress [168]. The co-treatment with α-lipoic acid and silver nanoparticles was also used to mitigate adverse effects of these nanoparticles in the treatment of pancreatic ductal adenocarcinoma. α-lipoic acid, in fact, reduced the production of reactive oxygen species (ROS) induced by the silver nanoparticles themselves, protecting non-malignant cells from oxidative stress [169].

### 4.2. α-Lipoic Acid and Gold Nanoparticle Renal and Hepatic Toxicity

Exposure to gold nanoparticles can induce hepatic oxidative damage and toxicities; it has been shown that the co-administration of α-lipoic acid with gold nanoparticles has protective effects, suppressing oxidative stress and inflammation [170]. Gold nanoparticles can also have nephrotoxic effects: α-lipoic acid has been demonstrated to reduce nanoparticle kidney damage, suggesting that combining natural antioxidants with gold nanoparticles may offer a potential strategy to prevent nanoparticle-induced toxicity [170,171,172].

### 4.3. α-Lipoic Acid and the Inhibition of Toxicity Induced by Other Types of Nanoparticles

α-lipoic acid exhibited hepatoprotective functions against zinc oxide nanoparticle toxicity, decreasing liver damage and the expression of metabolic disorder markers, which were up-regulated by the treatment with zinc oxide nanoparticles [173]. The co-administration of α-lipoic acid also significantly mitigated the cardiotoxic effects of zinc oxide nanoparticles by reducing serum cardiac injury markers, pro-inflammatory biomarkers, nitric oxide, and vascular endothelial growth factor levels, as well as cardiac calcium concentration and oxidative DNA damage, indicating its potential as s protective agent against ZnO-NP-induced cardiac tissue injury [174]. Moreover, α-lipoic acid displayed a therapeutic role after the exposure to lead and zinc oxide nanoparticles, attenuating the biochemical alterations in neurological, immunological, and male reproductive organs of rats, suggesting its potential as a protective agent against their adverse effects [175]. Cobalt nanoparticles can be generated from cobalt-containing implants and they can induce ferroptosis-like cell death through, among other things, increasing intracellular reactive oxygen species. Cobalt nanoparticle adverse effects have been mitigated by the co-administration of α-lipoic acid, which effectively counteracts the adverse effects induced by CoNPs, thus stabilising the cell’s antioxidant capacity and preventing ferroptosis-like cell death [176]. α-lipoic acid was also shown to be a promising hepatoprotective agent against copper-nanoparticle-induced oxidative damage; its administration mitigated hepatic damage, maintaining an antioxidant status [177]. 

## 5. Poly(α-Lipoic Acid)-Based Polymeric Nanoparticles: Insights and Perspectives

**Figure 5:**
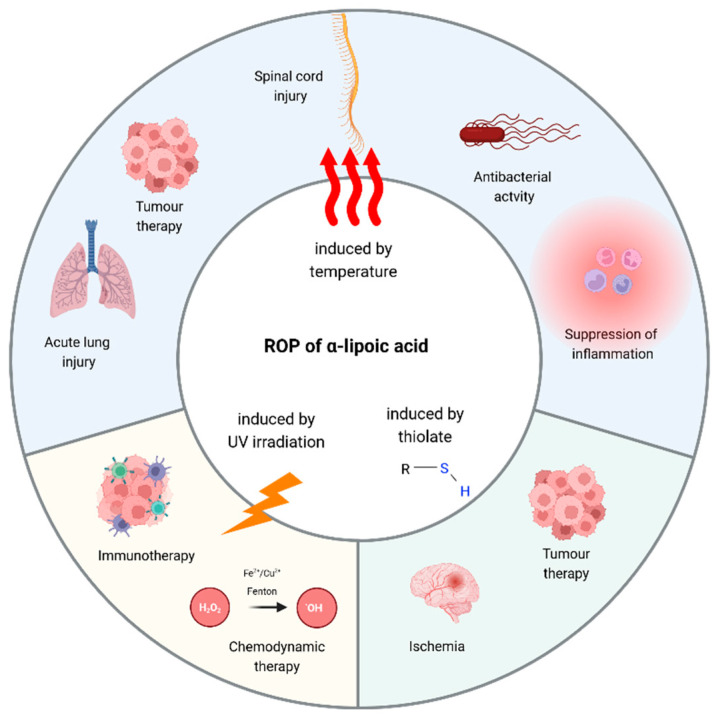
ROP of α-lipoic acid can be obtained thanks to three different strategies; by temperature, by irradiation, and by thiolate initiation. All these protocols were used to prepare poly(α-lipoic acid) nanoparticles, which were then tested for their abilities to fight various pathologies or pathological environments, and to enhance different types of therapies. Created with BioRender.com (accessed on 3 June 2024).

The 1,2-dithiolane ring contained in α-lipoic acid can undergo ring-opening polymerization (ROP) to produce poly(disulfide)s [178]. ROP [179] is the approach most used to propagate α-lipoic acid polymerization and obtain its cross-linking. ROP can be achieved by using different methods: elevated temperatures, photo-initiated radical polymerization, and thiolate compounds (see Figure 5). 

All these approaches have been used to produce nanoparticles based on the polymerization of α-lipoic acid. 

It is interesting to note how heat and light are often listed as factors that destabilise α-lipoic acid (see Introduction), but if used properly, they are good alternatives for achieving polymerization. On the other hand, ROP initiated using a thiolate monomer represents a promising approach due to the mild conditions it requires and the better control of the polymerization process [180]. The advantages and limitations of each strategy are summarised in Table 3.

### 5.1. Poly(α-Lipoic Acid) Nanoparticles Obtained by Thermal Polymerization

Poly(α-lipoic acid) nanoparticles, synthesised via thermal polymerization, were employed alongside various drugs to augment their anticancer efficacy. The general method applied in the studies outlined in this section involves polymerizing α-lipoic acid at a temperature above its melting point, then utilising the resulting poly(α-lipoic acid) to create the desired nanoparticles.

For instance, Yang et al. synthesised a PEGylated poly(α-lipoic acid) copolymer and assembled it into nanoparticles in aqueous solution. The nanoparticles showed the ability to load doxorubicin with high efficiency, and the doxorubicin-loaded nanoparticles were efficiently internalised in 4T1 cancer cells, where doxorubicin was released, displaying its antiproliferation ability. Moreover, these nanoparticles exhibited improved antitumoral efficacy in a mouse model, while causing fewer toxic effects on healthy organs [182]. The same nanoparticles were co-loaded with paclitaxel and doxorubicin and both were studied in a cellular and an animal model for osteosarcoma. The authors found that the dual-drug-loaded nanoparticles were effectively taken up by osteosarcoma cells, releasing drugs intracellularly in response to an acidic pH and the reductive environment of cancer cells. Moreover, the co-loaded nanoparticles exhibited improved biodistribution into the tumour in mice, resulting in a more effective inhibition of tumour growth compared to free drugs [183]. A similar strategy was used to develop pH-responsive nanovesicles from a PEGylated poly(α-lipoic acid) copolymer to deliver the chemotherapeutics doxorubicin and gefitinib for the treatment of ovarian cancer. The drugs were efficiently encapsulated and released intracellularly in a pH-responsive manner, inducing the apoptosis of cancer cells [184]. Liu and collaborators conjugated poly(α-lipoic) nanoparticles to combretastatin A4, a potent vascular disrupting agent, resulting in a specific accumulation of combretastatin A4 in tumour cells and in a prolonged apoptosis of cancer cells because of its long retention time [185]. Also, they used the same approach with the cytotoxic drug honokiol and another vascular disrupting agent, 5,6-dimethylxanthenone-4-acetic acid, obtaining a nanoparticle with high antitumor potency against a murine model of breast tumour [186].

Poly(α-lipoic acid) nanoparticles obtained thanks to elevated temperatures were also tested for their potential ability to treat spinal cord injury. The antibiotic minocycline was encapsulated in polymeric nanoparticles, and loaded with methylprednisolone, a drug used to inhibit inflammation after traumatic spinal cord injury. The combined nanoparticle was biocompatible and exhibited a potent anti-inflammatory activity, able to inhibit proinflammatory cytokine release in a microglia cell line treated with LPS [187]. 

The encapsulation of Venetoclax, a Bcl2 inhibitor, in polymeric nanoparticles has been demonstrated to enhance its effects when administered freely. It inhibits the production of pro-inflammatory cytokines in a murine model of lung injury by inducing the apoptosis of infiltrated neutrophils [188]. Also, the antioxidant properties of the natural compound quercetin were enhanced through encapsulation in polymeric nanoparticles. This encapsulation favoured its chemical stability and slow release, and the antioxidants properties of encapsulated quercetin have been shown to be improved with respect to its crystalline form in a rat model of acute liver injury [189]. 

Rhein (an anthraquinone with anti-inflammatory activity) and geraniol (a bioactive compound) were loaded onto poly(α-lipoic acid) acid nanoparticles, showing an enhanced antimicrobial activity against *Salmonella* infection, compared with free drugs. Additionally, co-loaded nanoparticles were also able to suppress the production of pro-inflammatory cytokines and to maintain the gut microbiota homeostasis [190]. With the aim of tackling *Salmonella* infection, characterised by the production of high levels of H_2_S, PEGylated α-lipoic acid was thermally polymerized and polymeric nanoparticles were loaded with the antibiotic compound ciprofloxacin. The nanoplatform, besides demonstrating excellent stability in vitro and biocompatibility toward mammalian cells, selectively delivered the antibiotic to the inflammation site, thus improving its efficacy [191]. 

### 5.2. Poly(α-Lipoic Acid) Nanoparticles Obtained by Thiolate-Initiated Polymerization

Matile and colleagues examined the reactivity of cyclic disulfides in disulfide-exchange polymerization. They concluded that α-lipoic acid’s disulfide bond length and the torsion angle of the thiolane ring exhibited the optimal reactivity to achieve optimal and controlled polymerization [192]. In most of the studies detailed in this paragraph, the process begins with the formation of nanoparticles containing α-lipoic acid, followed by the induction of polymerization using a thiolate initiator to yield poly(α-lipoic acid) nanoparticles.

Cross-linked α-lipoic acid nanocapsules using 1,4-dithiothreitol (DTT) thiolate have been employed to encapsulate two precursors of combretastatin (a compound displaying strong inhibitory activity on tumour cell growth) and a copper catalyst. Encapsulation ensured that the prodrug was not prematurely activated. Within the tumour tissue, the high concentration of GSH induces the breakdown of poly(α-lipoic acid) nanoparticles, releasing prodrugs and catalysts and inducing the death of only tumour cells [193]. The polymerization of α-lipoic acid was used to project nanoparticles based on a conjugate of lipoic acid and hyaluronic acid; these cross-linked nanoparticles were used to encapsulate and then deliver doxorubicin specifically to breast cancer and myeloma and leukaemia cells [194]. DTT was also used to induce the polymerization of α-lipoic acid contained in a dimeric surfactant-like amphiphilic molecule: this molecule can form nanoaggregates, due to the hydrophobic head of α-lipoic acid and the hydrophilic tail of gemini surfactant. Nanoaggregates were then stabilised thanks to α-lipoic acid polymerization and loaded with doxorubicin: the nanonetwork was selectively cytotoxic against cancer cells with respect to normal cells [195]. 

Mancin and collaborators showed that α-lipoic acid polymerization could be realised through a single-step synthesis using 1-octanethiol as initiator, producing cross-linked poly(α-lipoic acid) nanoparticles loaded with dye and protected by PEG directly from small molecule precursors [196]. Interestingly, they conducted a comparison on the long-term stability of nanoparticles synthesised with a thiolate initiator versus those formed without any initiators. The results highlighted that nanoparticles produced through thiol-initiated ROP resulted in the formation of more rigid and stable nanoparticles. Also, α-lipoic acid preserved its antioxidant activity within the nanoformulation, demonstrating in vitro efficacy in mitigating damages caused by post-ischemic reperfusion [197]. The potential of these nanoparticles as cardiomyocyte-targeted drug was validated in rats, revealing their specific accumulation in the heart without inducing any toxicity in cardiomyocytes. Furthermore, the nanoparticles were tested in vitro as a delivery system for nucleic acids in cardiomyocytes, demonstrating their ability to enter this specific cell type and release their cargo in the perinuclear area [198]. 

Conversely to the previous works described in this section, Gu et al. first induced the polymerization of α-lipoic acid using cysteine hydrochloride, and then prepared poly(α-lipoic acid) nanoparticles through ultrasonic emulsification. The newly obtained nanoplatform was employed for the delivery of docetaxel, demonstrating specific drug targeting in cancer cells and preventing unspecific drug release [199]. 

### 5.3. Poly(α-Lipoic Acid) Nanoparticles Obtained by UV light

In the case of irradiation-induced polymerization, as well, the majority of studies first involve the synthesis of nanoparticles containing α-lipoic acid, followed by its polymerization. 

A new pH-responsive nanodrug based on a form of camptothecin (an inhibitor of topoisomerase) was loaded into cross-linked R(+)-α-lipoic acid nanoparticles, enhancing the anticancer activity of camptothecin, stabilising the drug, and achieving a higher drug concentration in the HT29 tumour cells [200]. 

UV cross-linked R(+)-α-lipoic acid nanoparticles were used to anchor quaternary ammonium salts provided with long alkyl chains, which have the ability to destabilise cell membranes. Their link to α-lipoic acid nanoparticles ensured that their activity occurs only within the cell, following the dissolution of the nanocontainer due to the cytosolic redox environment. As a result, they exerted a selective cytotoxic activity only to the tumour environment [201]. 

Two α-lipoic acid monomers were modified, one with a disulfide Zn-coordinated dipicolylamine analogue, known to boost protein transfection efficiency, and the other with a guanidinium side chain, mimicking the structure of cell-penetrating peptides. The modified monomers were then polymerized by irradiation, and the new compound showed enhanced delivery efficacy for nucleic acids compared to commercial transfection reagents, demonstrating potential for both gene therapy and immunotherapy applications [202].

Chemodynamic therapy is a kind of therapy based on inducing high concentrations of hydroxyl radical (one of the most toxic radicals) from hydrogen peroxide through the Fenton or Fenton-like reaction which can be catalysed by iron-containing compounds. The high concentration of hydroxyl ions in turn induces lipid peroxidation and consequent cell death through ferroptosis. This type of therapy is highly specific but requires high concentrations of iron, which is toxic to non-tumour cells [203]. With the aim of specifically delivering iron into tumour cells, an iron-doped cross-linked α-lipoic acid nano-aggregate was developed. Inside the tumour cells the new nanocompound was able to self-supply H_2_O_2_ and accelerate Fe^3+^/Fe^2+^ conversion, thus increasing ROS levels [204]. For the same purpose, UV cross-linked α-lipoic acid nanoparticles were utilised to anchor copper, which can catalyse the Fenton reaction even at neutral or slightly acidic pH levels. By conjugating copper to α-lipoic acid nanoparticles, the authors achieved a nanoplatform capable of consuming less copper, facilitated by the ability of dihydrolipoic acid to reduce Cu^2+^ to Cu^+^, thus maintaining the production of hydroxyl radicals for a longer period compared to iron [205]. 

In another study, PSMA (prostate-specific membrane antigen) was cross-linked to poly α-lipoic acid nanoparticles, with a *trans*-cyclooctene functionalized lysine linker also added. In this way, the authors obtained a nanoplatform specific for prostate cancer cells, with pro-apoptotic activity given by the α-lipoic acid’s ability to inhibit Bcl2 and equipped with pre-targeting for tetrazine (which can selectively conjugate with *trans*-cyclooctene), thus enabling radiotherapy, suggesting a promising therapeutic approach for metastatic castration-resistant prostate cancer [206]. 

Poly(α-lipoic acid) nanoparticles were also employed to load indole-3-methanol, a natural compound capable of activating Phosphatase and tensin homolog (PTEN). PTEN is a well-known tumour suppressor, whose expression is found to be three times lower in triple-negative breast cancer cells. The designed nanoplatform delivered indole-3-methanol to tumour cells, where its PTEN-activating ability was enhanced by the co-presence of α-lipoic acid, leading to the specific apoptosis of tumour cells [207]. 

Interestingly enough, the polymerization of α-lipoic acid induced by irradiation has also been used to stabilise and finely control the size of gold nanoparticles. In this case, Cely-Pinto and co-authors first induced the polymerization of α-lipoic acid through irradiation; then, they mixed poly(α-lipoic acid) with tetralone (a photoinitiator for gold reduction) and HAuCl_4_, and irradiated the solution again; this way, they obtained stabilised gold nanoparticles with a diameter of 20–40 nm [208]. 

### 5.4. Harnessing Poly(α-Lipoic Acid) in Hydrogel Engineering

Hydrogels are three-dimensional networks of hydrophilic polymers capable of retaining large amounts of water within their structure. These materials exhibit a unique combination of properties, including high water content, softness, and flexibility, resembling natural tissues [209,210]. Hydrogels can be synthesised from a variety of polymers, including α-lipoic acid. 

For example, both a coenzyme salt polymer (poly(sodium α-lipoate)) and a poly(α-lipoic acid) were incorporated in an elastomer adhesive patch. The patch had hydrogen bonding cross-links between poly(α-lipoic acid) and poly(sodium α-lipoate), which prevented poly(α-lipoic acid) depolymerization. This design enabled the sustainable delivery of bioactive α-lipoic acid and provided durable adhesion to oral mucosal wounds due to the adhesive action of poly(sodium α-lipoate). In mouse and mini-pig models of oral ulcers, the adhesive patch showed promising therapeutic effects. In fact, it accelerated ulcer healing by regulating the inflammatory environment of damaged tissue, maintaining oral microbiota stability, and promoting faster re-epithelialization and angiogenesis, showing promising therapeutic properties for treating oral ulcers [211]. Qi et al. projected an elastic patch made of methacrylated gelatin in combination with a poly(α-lipoic acid). Also, in this case, the crosslinking between poly(α-lipoic acid) and the methacrylated gelatin prevented poly(α-lipoic acid) depolymerization and slowed down its dissociation in water, enabling durable adhesion to oral periodontal tissue and the continuous release of bioactive small molecules in periodontitis wounds [212]. Hydrogels of poly(α-lipoic acid-*co*-sodium lipoate) were obtained by inducing the polymerization of the α-lipoic acid by heating in the presence of sodium bicarbonate. These green hydrogels maintained the ROS scavenging ability and they have been demonstrated to reduce inflammation in a rat spinal injury model, promoting the functional recovery of the spinal cord [213]. Hydrogels comprising poly(α-lipoic acid) and poly(ethylene glycol) were utilised as drug delivery vehicles to encapsulate doxorubicin, which was shown to be selectively released within the tumour microenvironment due to the presence of disulfide bonds in the hydrogel and the reducing conditions found in the tumour [214]. Additionally, a multifunctional supramolecular polymer hydrogel was engineered by merging gold nanostars with insulin through the ring-opening polymerization of α-lipoic acid. This hydrogel demonstrated the capacity to expedite the healing process of diabetic wounds infected by *S. aureus*, showcasing remarkable antibacterial efficacy under near-infrared irradiation (attributed to gold nanostars) and redox balance regulation (attributed to α-lipoic acid) [215]. 

Pluronic F127 has gained attention in the development of wound healing hydrogel due to the possibility of easily adjusting the phase transition temperature. In fact, F127 undergoes a sol–gel thermo-reversible transition within the range of room temperature to physiological temperatures. However, F127 hydrogel rapidly dissolves in physiological conditions, thereby limiting their efficacy. The functionalization of the copolymer was investigated as a strategy to target this drawback. 

For instance, the thiol/disulfide exchange reaction was employed to produce an injectable self-healing cross-linked hydrogel. α-lipoic acid-functionalized PEG reacted with thiol-modified F127 to form a dynamic thermoresponsive hydrogel with a rapid sol–gel transition ability at body temperature [216]. Also, α-lipoic acid was linked to Pluronic F127, resulting in a co-polymer that self-assembled in micelles. Subsequently, the hydrogel formation was obtained by the photo-induced cross-linking of α-lipoic acid. The hydrogel exhibited biocompatibility, optimal mechanical properties, and temperature-dependent swelling properties, and provided the release of bovine serum albumin in a reducing environment [217]. A similar approach was used by Wang et al., who conjugated α-lipoic acid to Pluronic F127 to create an injectable tissue-adhesive hydrogel. The exposure of the hydrophobic dithiolane ring of α-lipoic acid facilitated the formation of bigger micelles compared to F127 alone, promoting the hydrophobic aggregation. Moreover, α-lipoic acid was used to enhance the biocompatibility by preventing the potential toxicity of the aldehyde groups of F127. The micelles were combined with Ce^3+^/tannic acid/ulinastatin nanoparticles with LPS- and ROS-scavenging activity to obtain a wound-healing-promoting hydrogel. The nanohydrogel demonstrated excellent injectability and tissue adherence at physiological temperatures. Moreover, upon UV irradiation, the initially semisolid hydrogel solidified, providing tight tissue adherence. The application of the nanohydrogel on wounds suppressed inflammation and facilitated the healing process [218]. Eventually, an injectable hydrogel with near-infrared, antioxidant, and antiinflammatory properties was developed, incorporating α-lipoic acid-modified palladium nanoparticles into a sodium alginate hydrogel crosslinked with calcium ions [219].

## 6. Conclusions and Future Perspectives

α-lipoic acid has been extensively studied as a dietary supplement, with a current high impact and growing market. Moreover, it has been investigated in different therapeutic contexts, showing encouraging outcomes in both preclinical and clinical studies.

However, α-lipoic acid use has faced challenges, such as sensitivity to external factors, instability, and poor bioavailability, which affect its pharmacokinetic and pharmacological profile. Nevertheless, certain fundamental aspects are still debated and poorly understood, such as the impact of each enantiomer in the therapeutic activity and the paradoxical pro-oxidant activity despite its being a well-established antioxidant. Thus, additional investigation should be undertaken to elucidate these issues. 

Nanotechnology has emerged as a promising strategy to target these limitations and offers the possibility of an efficient formulation of α-lipoic acid. Furthermore, α-lipoic acid represents a promising scaffold for derivatisation and is used in the design of delivery nanoplatforms for different drugs and therapeutic areas. This review highlights numerous ways in which research on α-lipoic acid and nanotechnology has sought to address some of the challenges encountered when working with this compound. Although nanotechnological platforms incorporating α-lipoic acid have shown promise, many studies have focused solely on investigating the effects of nanoformulated α-lipoic acid at low concentrations or short exposure times, not considering the side effects observed with prolonged exposure to α-lipoic acid alone. Furthermore, the potential long-term side effects of the materials used to encapsulate α-lipoic acid (such as surfactants and copolymers) have often not been considered, and few studies provide data on the pharmacokinetic and pharmacodynamic properties of the new nanoplatforms. Further data also need to be obtained regarding the chemical degradation of the new nanoplatforms once in circulation and how they are eliminated from the body. Finally, many studies have been conducted using cellular models, while few studies utilise disease models in small animals, highlighting the need for further studies, including clinical trials testing nanoformulations of α-lipoic acid, to validate their effectiveness as formulations and delivery systems for α-lipoic acid.

## Figures and Tables

**Table 3:** Advantages and limitations of the most common methods used to synthesise poly(α-lipoic acid) nanoparticles [181].

Method	Mechanism	Advantages	Limitations
Thermal polymerization	Free radical polymerization	Solvent-free bulk conditions Rapid above the melting point	Potential degradation Uncontrollable (often results in a mixture of poly(α-lipoic acid) and cyclic monomers)
Thiolate-initiated polymerization	Thiol–disulfide exchange	Mild conditions Controllable (homogeneity) Rapid	Stability of the thiolate initiator Control of temperature and light exposure
Photo-initiated polymerization	Free radical polymerization	Mild conditions	Potential degradation Uncontrollable (often results in a mixture of poly(α-lipoic acid) and cyclic monomers)
